# In Vitro and In Vivo Antibacterial and Antibiofilm Activity of Zinc Sulfate (ZnSO_4_) and Carvacrol (CV) Alone and in Combination with Antibiotics Against *Pseudomonas aeruginosa*

**DOI:** 10.3390/antibiotics14040367

**Published:** 2025-04-01

**Authors:** Melika Moradi, Effat Abbasi Montazeri, Sirous Rafiei Asl, Ali Pormohammad, Zahra Farshadzadeh, Dian Dayer, Raymond J. Turner

**Affiliations:** 1 Infectious and Tropical Diseases Research Center, Health Research Institute, Ahvaz Jundishapur University of Medical Sciences, Ahvaz 6135715794, Iran; melika.moradi1@ucalgary.ca (M.M.); farshadzadeh-z@ajums.ac.ir (Z.F.); 2 Department of Microbiology, Faculty of Medicine, Ahvaz Jundishapur University of Medical Sciences, Ahvaz 6135715794, Iran; 3 Department of Biological Sciences, Faculty of Science, University of Calgary, Calgary, AB T2N 1N4, Canada; ali.pormohammad@ucalgary.ca; 4 Cancer, Environmental and Petroleum Pollutants Research Center, Ahvaz Jundishapur University of Medical Sciences, Ahvaz 6135715794, Iran; rafieiasl-s@ajums.ac.ir; 5 Alimentary Tract Research Center, Ahvaz Jundishapur University of Medical Sciences, Ahvaz 6135715794, Iran; 6 Cellular and Molecular Research Center, Medical Basic Sciences Research Institute, Ahvaz Jundishapur University of Medical Sciences, Ahvaz 6135715794, Iran; dayer-d@ajums.ac.ir

**Keywords:** *Pseudomonas aeruginosa*, biofilm-associated infections, plant-based natural compounds, zinc, carvacrol, combination effects, mouse lung infection

## Abstract

**Background/Objectives**: Biofilm-embedded bacteria, such as *Pseudomonas aeruginosa (P. aeruginosa)*, are highly resistant to antibiotics, making their treatment challenging. Plant-based natural compounds (PBCs) and metal(loid)-based antimicrobials (MBAs) are promising alternatives. This study evaluated the minimum inhibitory concentration (MIC), minimum bactericidal concentration (MBC), and synergistic effects of zinc sulfate (ZnSO_4_), carvacrol (CV), and antibiotics (ciprofloxacin [CIP], tobramycin [TOB], and azithromycin [AZM]) against *P. aeruginosa* PAO1. **Methods**: The MIC and MBC of ZnSO_4_, CV, and antibiotics were determined using a 96-well plate method. Cytotoxicity was assessed via MTT assay. Fractional inhibitory concentration (FIC), fractional bactericidal concentration (FBC), minimal biofilm inhibition concentration (MBIC), and minimum biofilm eradication concentration (MBEC) indices were calculated for each combination of agents. Checkerboard assays identified interactions, and the effectiveness of combinations was further evaluated in a mouse chronic lung infection model with treatments delivered intratracheally, intraperitoneally, and orally. **Results**: TOB had the lowest MIC and MBC values, proving most effective against *P. aeruginosa* PAO1. Strong synergy was observed with CV + ZnSO_4_ (CV + Zn) combined with CIP, CV with CIP, and CV + Zn with TOB, as indicated by low FIC indices. CV + Zn with TOB and CV with TOB had low FBC indices, while CV + Zn with AZM showed antagonism. In vivo, intratracheal TOB + CV + Zn reduced lung inflammation and tissue involvement, yielding the best histopathological outcomes. The MIC of CIP and TOB was reduced 5-fold and 4-fold, respectively, when combined with CV + Zn. **Conclusions**: CV + Zn demonstrated strong synergistic effects with antibiotics and effectively managed *P. aeruginosa* lung infections in mice. These findings highlight its potential as an innovative therapy for biofilm-associated infections.

## 1. Introduction

*Pseudomonas aeruginosa* is an opportunistic Gram-negative bacteria that is responsible for 10% of all hospital-acquired infections [1]. Immunocompromised patients and individuals with underlying disease or weakened immune systems are vulnerable to *P. aeruginosa* infections [2,3]. This bacterium causes various community- and nosocomial-associated infections, including burn sepsis, nosocomial and ventilator-associated pneumonia, chronic obstructive pulmonary disease (COPD), urinary tract infections, meningitis, wound infections, and infection of the conjunctiva [4].

*P. aeruginosa* can perpetuate itself by finding a favorable ecological niche in people with chronic airway inflammation. This bacterium is the main cause of chronic bronchial infection in people with cystic fibrosis (CF) and COPD [5]. It has been determined that *P. aeruginosa* is present in the respiratory secretions of individuals with long-lasting asthma, frequent exacerbations, and concomitant bronchiectasis. Therefore, it is one of the main targets in the treatment of patients with respiratory tract infections [6].

The World Health Organization (WHO) has declared *P. aeruginosa* as one of the 12 drug-resistant bacteria of concern. Currently, this bacterium is considered a main threat to human health [7]. In general, penicillin, cephalosporins, carbapenems, aztreonam, fluoroquinolones, and aminoglycosides are the commonly used antibiotic treatments of infections caused by *P. aeruginosa* in healthcare settings [8]. Patients with conditions such as CF frequently require antibiotics as part of their daily treatment regimen to manage *P. aeruginosa* infections, which often leads to morbidity and mortality. Treatment often involves a combination of inhaled, oral, and intravenous (IV) antibiotics, depending on disease severity and bacterial resistance [9,10]. However, there is no consensus on the optimal combination, dosage, or duration of treatment [11]. Inhaled antibiotics, such as tobramycin, are preferred for chronic *P. aeruginosa* infections, as they deliver high local drug concentrations directly to the lungs while reducing the need for IV treatments and minimizing systemic toxicity [12]. IV antibiotics, such as ciprofloxacin, are administered during acute exacerbations to achieve rapid bacterial clearance [13]. Oral macrolides, particularly azithromycin, are used adjunctively due to their anti-inflammatory properties and ability to disrupt biofilm formation, interfering with *P. aeruginosa* adherence to epithelial cells, reducing bacterial persistence [13,14,15]. Although these treatment recommendations are based on the best available evidence, medical centers may tailor therapies based on their clinical experience and individual patient needs [11]. While early antibiotic treatment may resolve symptoms and clear bacteria, chronic infections frequently develop [16]. Over time, *P. aeruginosa* in the infection undergoes considerable genetic adaptations [17], enabling it to persist in the airways, evade the host immune response, and resist antibiotic treatment [9,10]. The emergence of intrinsic and acquired resistance mechanisms in *P. aeruginosa* has made the management of these infections increasingly challenging in clinical settings [18]. Despite these strategies, *P. aeruginosa* exhibits extensive resistance mechanisms, highlighting the need for alternative or adjunctive treatments to enhance therapeutic outcomes [11,19].

Biofilm formation is an antibiotic resistance mechanism among bacteria [18,20]. Biofilm is an aggregated and organized community of microcolonies embedded in a self-generated extracellular matrix (ECM) [21]. Treatment and eradication of infections caused by *P. aeruginosa* in the biofilm state are very difficult [22]. It is well known that biofilms of *P. aeruginosa* demonstrate up to 1000-fold increased resistance in comparison to their planktonic counterparts [23]. *P. aeruginosa* is the main cause of lifelong chronic airway infections in adult patients with CF that are resistant to the host immune system and antibiotic therapy [24]. *P. aeruginosa*’s biofilm growth is largely responsible for the chronic, non-invasive, and drug-resistant nature of chronic infections [25].

According to the WHO reports, new antimicrobial compounds are urgently required to improve the efficacy of currently used antibiotics to kill biofilm-embedded bacteria [19,26]. The use of combination therapy is considered one of the strongest strategies for the treatment and prevention of antibiotic-resistant infections. In previously published studies, plant-based natural compounds (PBCs) and metal(loid)-based antimicrobials (MBAs) have been explored as potential alternatives to treat antibiotic-resistant infections [18,19,27]. MBAs are commercially available, and several are already used in healthcare, industry, and agriculture and actively researched [27,28,29,30]. Zinc plays a critical role in prokaryotes and eukaryotes, ranging from structural to catalytic [31]. Zinc is one of the essential minerals that play an important role in different biological activities in humans [32], supporting mucociliary clearance and enhancing the protective function of the respiratory epithelium [33,34]. Zinc influences various cellular processes but can also exert antimicrobial effects [31]. Zinc ions can inhibit bacterial glycolysis and increase proton permeability across bacterial membranes, disrupting cellular homeostasis [35]. Additionally, zinc has demonstrated antibiofilm properties by interfering with bacterial adhesion and biofilm formation [36]. Delivering optimal zinc levels may help reduce the risk of bacterial co-infections and support respiratory health [33,34].

PBCs have several drug-like features, such as antimicrobial, antibiofilm, antioxidant, emulsifying, thickening, film-forming, and prebiotic activity [18,37]. PBCs can be relatively inexpensive products that may also have anti-inflammatory and anticancer features. Additionally, it has been demonstrated that PBCs can boost the immune system [19,38].

In recent years, more than 50 different PBCs have been recognized that have natural antibacterial activity [38]. It has been shown that PBCs have limited toxicity and side effects and have good potential to formulate novel antimicrobials. Carvacrol is the main component of oregano oil and thyme and exhibits antibacterial, anti-inflammatory, and antioxidant properties [39]. Additionally, carvacrol possesses a range of other biological activities [40], including antifungal [41], antitumor, antimutagenic, analgesic, anti-hepatotoxic, cardioprotective, and antiparasitic effects [42,43]. The combination of PBCs and conventional antibiotics has demonstrated synergistic antimicrobial effects [19]. Several studies have revealed that the combination can enhance the effectiveness of traditional antibiotics against Gram-negative bacteria [18,19,23,27].

In this study, we investigated the antimicrobial and antibiofilm effects of carvacrol (CV) (as a PBC), zinc sulfate (ZnSO_4_) (as an MBA), and a combination of these two (CV + ZnSO_4_) alone and in combination with ciprofloxacin (CIP), tobramycin (TOB), or azithromycin (AZM) against the *P. aeruginosa* reference strain PAO1. Moreover, we also evaluated the efficacy of the CV + Zn antimicrobial formulation on *P. aeruginosa* pneumonia in a mouse model.

## 2. Results

### 2.1. Selected Agents for Experiments

**Figure 1:**
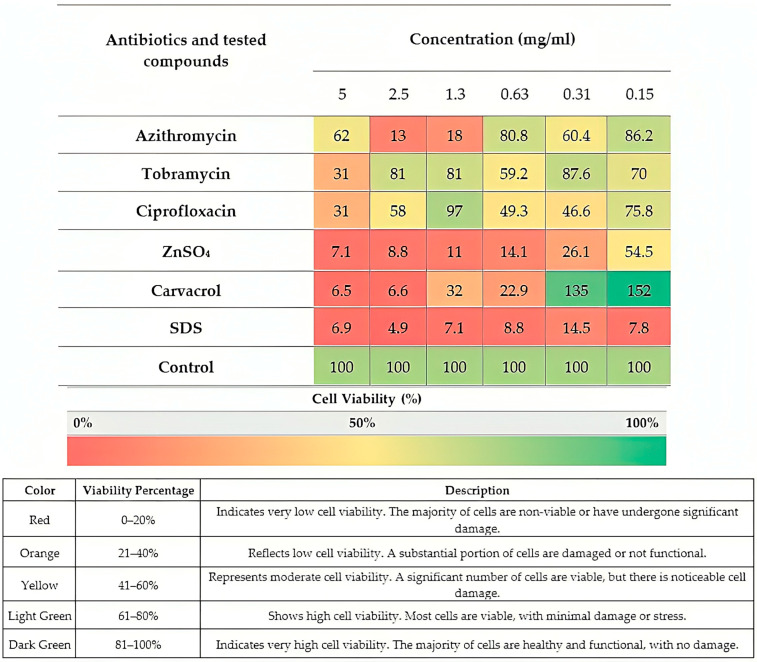
Cytotoxicity assessment of antimicrobial agents after 24 h exposure. Heat map displaying the viability percentage of MRC-5 lung fibroblast cells exposed to varying concentrations of antimicrobial agents.

For the selection of metal-based antimicrobial and plant-based compounds to explore, we used our previously evaluated antimicrobial activity information from a number of metals [28] and natural plant compounds [20]. From this information, the goal was to identify the best antimicrobial metal and PBC to explore for combined effects with low cytotoxicity. These studies suggested ZnSO_4_ and CV. An MTT tissue culture assay revealed that ZnSO_4_ exhibited moderate cytotoxicity (Figure 1) and had a dose-dependent reduction in cell viability at higher concentrations, consistent with previous studies reporting cytotoxic and genotoxic effects of Zn^2^⁺ in eukaryotic cells at concentrations ranging from 25 to 300 µM [44]. The cytotoxicity data in Figure 1 also show that CV has a moderate cell toxicity. Both of our choices are more cytotoxic than the antibiotics tested but less than other MBAs or PBCs, so we considered that combinations should still be explored. Therefore, ZnSO_4_ and CV were selected, and we investigated the antimicrobial and antibiofilm effects of CV (as a PBC), ZnSO_4_ (as an MBA), and a combination of two compounds (CV + Zn) alone and in combination with the antibiotics AZM, CIP, and TOB against the *P. aeruginosa* reference strain PAO1.

### 2.2. Antimicrobial Synergy Investigation—Planktonic Cultures

Our study aimed to examine the MIC and MBC of ZnSO_4_ and CV, in addition to conventional antibiotics (AZM, CIP, and TOB), against the *P. aeruginosa* reference strain PAO1. The antimicrobial efficacy of CV and ZnSO_4_ against the bacteria ranged from 2.5 to 20 mg/mL (Table 1). The most effective antibacterial agent on its own for *P. aeruginosa* was TOB, with CIP coming in second, while AZM demonstrated the highest MIC values among these antibiotics. The results indicate that TOB and CIP exhibit strong bactericidal activity against the PAO1 strain examined.

The antibacterial effects of CV, ZnSO_4_, and a combination of two compounds (CV + Zn) were evaluated in combination with AZM, CIP, and TOB against the *P. aeruginosa* reference strain PAO1. The best bacteriostatic synergistic effects were observed by combining CV + Zn with CIP, CV with CIP, and CV + Zn with TOB, as evidenced by the FIC index of 0.2, indicating a strong synergy (Table 2). In addition, the combination of CV with TOB, ZnSO_4_ with AZM, and ZnSO_4_ with CIP resulted in an FIC index of 0.4, indicating synergy. The results showed that the combination of CV with ZnSO_4_, CV + Zn with AZM, and CV with TOB exhibited FIC values of 1, suggesting a neutral interaction between the antibiotics. When CV + Zn was combined with AZM, the resulting FIC index of 2.2 indicated an antagonistic interaction.

By evaluating the FBC, we could see bactericidal synergies (Table 2). The combinations of CV + Zn with TOB and CV with TOB showed strong synergy. Moreover, the combined effect of ZnSO_4_ with TOB demonstrated synergy. Furthermore, the combinations of CV + Zn with CIP and CV with CIP were both bactericidally synergistic. Again, CV + Zn with AZM was antagonistic. In addition, the combination of ZnSO_4_ with AZM exhibited an antagonistic relationship.

### 2.3. Antimicrobial Synergy Investigation—Biofilms

CV + Zn was methodically combined with each of the three antibiotics to test their effectiveness against *P. aeruginosa* PAO1 in a biofilm. Table 3 presents the patterns of synergism for eradicating biofilms, along with the corresponding FBEC values. Table 3 provides the FBEC ranked from highest to lowest (highest synergism combinations) for the convenience of the reader. The combination of CV + Zn + CIP was found to be the most effective with excellent synergistic activity in terms of biofilm eradication. The use of CV and ZnSO_4_ alone with CIP, as well as their combined application (CV + ZnSO_4_), resulted in an FBEC index ranging from 1 to 2.5, indicating relatively lower effectiveness in eradicating biofilms compared to the CV + Zn + CIP combination.

The MIC of ciprofloxacin alone for bacterial growth inhibition was measured to be 0.001 mg/mL. When CV + Zn was combined with CIP, the MIC decreased to 0.0002 mg/mL. Therefore, the combination of CV + Zn with CIP reduced its effective concentration by 5-fold compared to CIP alone. A similar trend emerged with TOB, where the concentration required for effectiveness decreased by approximately 4-fold when compared to TOB alone. A consistent decreasing pattern in the bactericidal effect (MBC) of all three antibiotics was observed, particularly for AZM and TOB, with a significant 12- and 8-fold decrease, respectively.

The MBEC for the combination of CV + Zn and CIP alone was 10 and 0.03 mg/mL, respectively. However, when CV + Zn was combined with CIP, the effective concentration for CV + Zn plus CIP decreased to 0.156 and 0.007 mg/mL, resulting in a 64- and 4-fold reduction in concentration.

### 2.4. In Vivo Respiratory Infection in a Murine Model

#### 2.4.1. Pulmonary Bacterial Clearance

**Figure 2:**
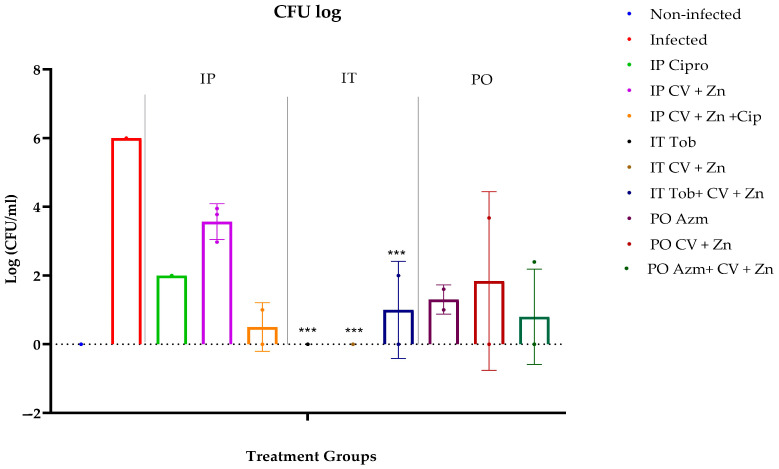
CFU analysis of CV + Zn and antibiotic administration routes on pulmonary *P. aeruginosa* PAO1 clearance. Mode of antimicrobial delivery: IT (intratracheal inhalation), IP (intraperitoneal injection), and PO (oral administration). The red graph indicates the infected control group without treatment. Mice were infected with approximately 5 × 10^5^ CFU PAO1 instilled intratracheally, initiating antibacterial treatments 24 h post-infection. Each treatment was administered once daily for three days: TOB (IT), CIP (IP), AZM (PO), and CV + Zn alone and in combination with antibiotics via IT, IP, and PO routes. CFU counts were assessed from lung homogenates, with results calculated logarithmically relative to controls. Data points are represented as means (*n =* replicates). Error is shown in ±standard deviation (SD). Statistics were performed using one-way ANOVA, comparing treatments to infected control. *p* values are <0.001 = ***.

At the end of the treatment period, the infected control group exhibited the highest bacterial load and severe lung infection, with lung tissue involvement and high pneumonitis scores. Among the treatments, IT administration of CV + Zn and TOB demonstrated the most effective reduction in bacterial load in the mouse lungs (Figure 2).

#### 2.4.2. Host Immune Response

**Figure 3:**
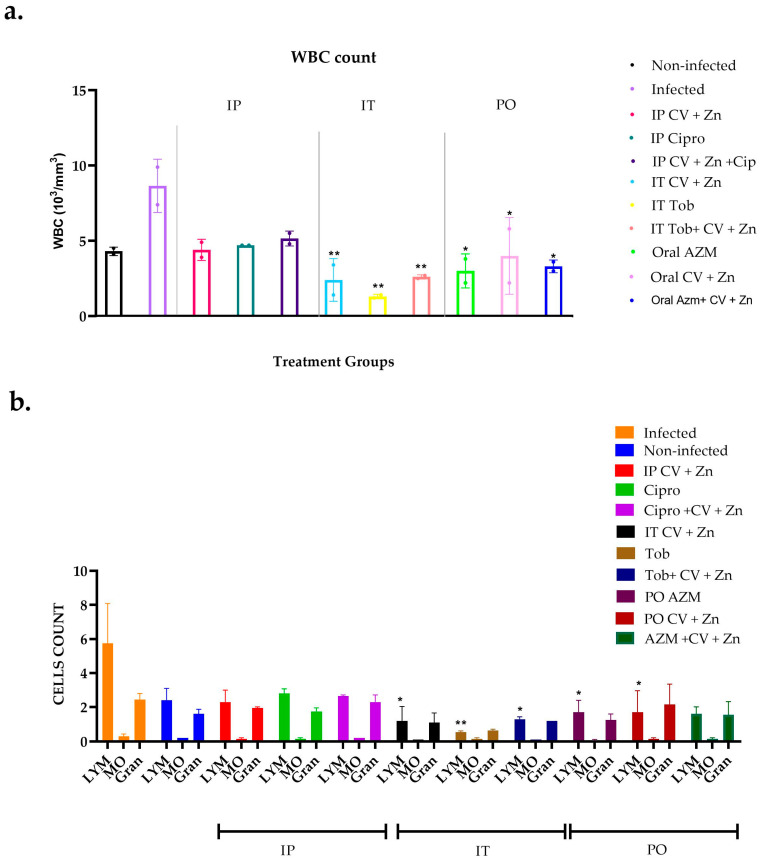
Host immune response evaluating white blood cell (WBC) response. (**a**) Total WBC; (**b**) subpopulation (LYM: lymphocytes; Mo: monocyte; Gran: granulocytes) of WBC. Analysis of CV + Zn and antibiotic administration routes on pulmonary *P. aeruginosa* PAO1 clearance in a murine model. Data points are represented as means (*n* = replicates). Error is shown in ± standard deviation (SD). Statistics were performed using one-way ANOVA, comparing treatments to infected control. *p* values are, between 0.001 and 0.01 = **, and between 0.01 and 0.05 = *.

Analysis of lung homogenates revealed a substantial increase in white blood cell (WBC) counts in the infected group compared to the treated groups. Treatments administered via the IT route demonstrated effective infection control, while PO AZM combined with CV + Zn produced moderate results. The inflammatory response in the infected group was marked by neutrophil counts and elevated lymphocytes. The IT group showed the most reduction in WBC subpopulations, while the distribution of key WBC subpopulations was similar between non-infected and oral-challenged mice, with minor lymphocytosis observed in the TOB group. For detailed data, refer to Figure 3.

#### 2.4.3. Histopathological Analysis

**Figure 4:**
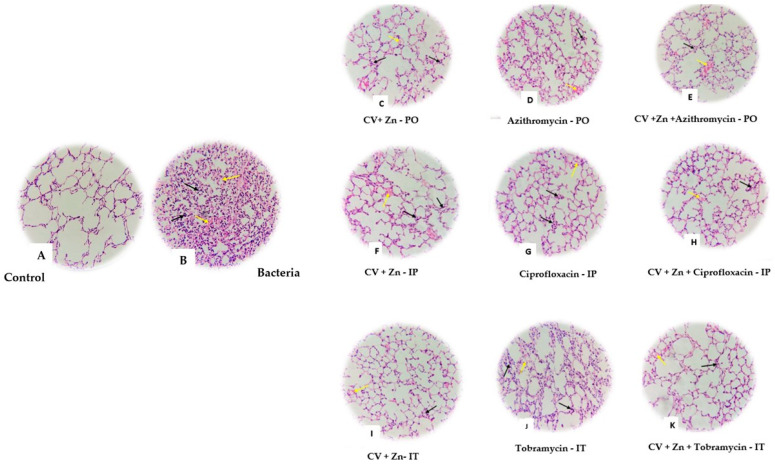
H&E staining. In the non-infected control article, no special damage was observed in terms of histopathology (**A**). In the group receiving bacteria (**B**) and other groups receiving drugs along with bacteria in intratracheal (IT), peritoneal (IP), and oral (PO) form (**C**–**K**), pneumonia with bleeding (yellow arrow) and infiltration of inflammatory cells (black arrow) can be seen. Magnification 40×.

The non-infected control group showed no lung tissue involvement, maintaining an 80% alveolar air area (Figure 4). The bacteria group exhibited severe pathology with 80% lung tissue involvement and high pneumonitis scores (Table 4). Treatments with TOB combined with CV + Zn and CV + Zn alone, administered via IT and PO routes, as well as AZM combined with CV + Zn administered orally, resulted in lower lung tissue involvement and fewer periluminal infiltrates compared to single treatments. While antibiotics alone resulted in moderate to high lung tissue involvement, the combinations of antibiotics with CV + Zn resulted in reduced lung involvement compared to single treatments. Notably, the combination of TOB and CV + Zn via IT administration yielded the lowest lung tissue involvement (5%) and minimal periluminal infiltrates (Figure 4 and Table 4).

## 3. Discussion

The use of existing antibiotics and new antimicrobial alternatives in a synergistic manner to create a novel effective combination is promising and is of interest for several reasons: (1) bacterial cell viability can be completely eliminated, and any recovery can be prevented; (2) the development of future resistance decreases by mixing antimicrobials with different cell targets, as the probability of simultaneous mutants in two genes resistant to both antimicrobials is extremely low; and (3) the effective concentration of antibacterial agents can be reduced by mixing them, which results in decreased toxicity and side effects for the host [19,37,38].

Plant-based natural compounds (PBCs) and metal(loid)-based antimicrobials (MBAs) [28,29] are considered potential antimicrobials for preventing and eradicating antibiotic-resistant infections. In the present study, we considered the use of these to synergistically activate present-day antibiotics for potential treatment against *P. aeruginosa* infections. Results of the broth microdilution assay revealed that among MBAs and PBCs, ZnSO_4_ and CV had some of the lowest MIC and MBC values against *P. aeruginosa*. Moreover, the MTT assay revealed that ZnSO_4_ and CV had the lowest cell cytotoxicity. Therefore, these two compounds can be combined at their best concentrations to generate a novel antimicrobial formulation.

Then, we evaluated this mixture alone and in combination with the antibiotics AZM, CIP, and TOB against the *P. aeruginosa* reference strain PAO1 for synergies and efficacy in a mouse model. Our selection of CIP, AZM, and TOB in this study was based on their clinical relevance in CF therapy and their common routes of administration in clinical settings, toxicity, and treating respiratory infections, including cystic fibrosis (CF)-associated bacterial infections. Each of these antibiotics is commonly used in different routes of administration, which influences their distribution, efficacy, and potential for combination therapy [11]. While both AZM and TOB target bacterial protein synthesis, their mechanisms and clinical applications differ; TOB binds to the 30S ribosomal subunit, causing misreading of mRNA and bactericidal effects, and AZM, although targeting the 50S ribosomal subunit [45], is not primarily used as a bactericidal agent in respiratory infections. Instead, it is prescribed for its anti-inflammatory and anti-biofilm properties, which improve lung function and reduce exacerbations [46]. Furthermore, aminoglycosides and fluoroquinolones exhibit concentration-dependent pharmacodynamics, so increased drug concentrations delivered by aerosol may translate into augmented antibacterial activity; thus, inhalation of antibacterials may augment efficacy and reduce systemic toxicities, but there is a risk of higher allergy in patients in inhalation therapies [11,47].

In the present study, TOB had the lowest MBC and MIC values and was considered the most effective antibacterial agent against the *P. aeruginosa* strain *PAO1.* Our study revealed that combining CV + Zn with CIP, CV with CIP, and CV + Zn with TOB showed the best bacteriostatic synergistic effects. Moreover, the combinations of CV + Zn with TOB and CV with TOB showed strong synergy for bactericidal outcome. In contrast, we showed that CV + Zn when combined with AZM resulted in an antagonistic interaction.

Several studies have investigated the antimicrobial and antibiofilm effects of CV alone and in combination with other antimicrobial materials against Gram-positive or Gram-negative bacteria. Hasanvand et al. investigated the antibacterial activity of CV + ethanol on *P. aeruginosa* and *S. aureus* isolates. They showed that carvacrol had more antibacterial effects against *P. aeruginosa* and *S. aureus* isolates. Their findings revealed that the optimal concentration of CV + ethanol against *P. aeruginosa* and *S. aureus* was 64 μL/mL and 8 μL/mL, respectively [48]. In a previous study from our group, the antibacterial and antibiofilm activity of several PBCs compared with CIP and gentamicin against different bacteria was explored. Among 15 different PBCs, CV, tetrahydrocannabinol, cinnamaldehyde, and cannabidiol had the most promising antibacterial and antibiofilm effects [18].

Carvacrol (2-methyl-5-(1-methylethyl)phenol) is a hydrophobic monoterpene phenol present in essential oils of different plants, such as *Origanum vulgare*, *Thymus vulgaris*, *Lepidium flavum*, and *Citrus aurantium* var. *bergamia* Loisel [49]. CV has a wide range of biological activities, including antibacterial, antiviral, antifungal, antioxidant, and anticarcinogenic properties [49,50]. Carvacrol primarily targets bacterial cell membranes, disrupting their integrity and causing cell death by releasing intracellular contents. Scanning electron microscopy (SEM) further confirms this membrane damage [51]. CV has been shown to prevent and inhibit the initial step of biofilm formation and biofilm maturation stages [50]. It is stated that CV can reduce the risk of infections associated with antimicrobial-resistant pathogens [52]. CV was found to have no cytotoxic effect on human cells when tested at a maximum concentration of 90 mg/mL [42,53,54,55]. It has been shown that CV can reduce cytotoxicity and inflammatory mediators, decrease lung inflammation, improve respiratory symptoms, and increase pulmonary function test (PFT) values by reducing cell necrosis and apoptosis in patients with COPD and asthma [51,54,56,57]. The United States Food and Drug Administration (USFDA) granted approval for the use of CV as a flavoring agent in foods under the “Everything Added to Food in the United States” (EAFUS) status [58]. The high abundance and safety of CV make for an ideal PBC to exploit as a pharmaceutical drug.

Zinc plays a vital role in cellular growth, differentiation, and metabolism. Zinc deficiency can inhibit childhood growth and reduce resistance to infections [58]. It is responsible for regulating carbohydrate and lipid metabolism as well as the function of the reproductive, cardiovascular, and nervous systems. Zinc is crucial in regulating the immune system, as it affects the proliferation, differentiation, maturation, and function of leukocytes and lymphocytes [59]. Changes in zinc levels have an impact on the immune response, resulting in an increased risk of inflammatory and infectious diseases, including acquired immune deficiency syndrome, measles, malaria, tuberculosis, and pneumonia. Alteration of zinc status increases the susceptibility to inflammatory and infectious diseases, including pneumonia, acquired immune deficiency syndrome, tuberculosis, measles, and malaria [59,60].

The role of zinc as an essential trace element in many physiologic, enzymatic, and structural functions is well established [61]. Zinc has been shown to help reduce lung inflammation by limiting neutrophil recruitment and activity, thereby minimizing lung damage. Its anti-inflammatory and immune-modulatory properties also show potential in alleviating symptoms, reducing cytotoxicity, and decreasing cell necrosis and apoptosis in patients with respiratory diseases such as asthma and COPD [59,62,63]. Moreover, the clinical symptoms of COVID-19 can be improved in patients with SARS-CoV-2 infection by orally administering large doses of zinc salts. Zinc supplementation has been shown to reduce antibiotic usage and infection frequency in patients with cystic fibrosis and respiratory tract infections [60]. The mechanism of action is likely through modulating some of the viral enzyme activity [64].

Here, we showed that the sole use of CV + Zn had a relatively low effectiveness in eradicating biofilms. However, the combination of CV + Zn + CIP demonstrated the most promising result of all combinations in terms of biofilm eradication. This finding suggests a high level of synergism between the CV + Zn + CIP and indicates a high level of effectiveness in eliminating biofilms. Asadi et al. examined the synergistic antibacterial and antibiofilm effects of CV in combination with cefixime against *E. coli*. They found that CV with FIC index = 0.5 had a synergistic interaction with cefixime against *E. coli.* They showed that the combination of CV and cefixime inhibited biofilm formation at MIC/2, MIC/4, and MIC/8 [65]. Puca et al. investigated the activities of a carbonic anhydrase inhibitor (CV), amoxicillin (AMX), and a urease inhibitor (SHA) alone and in combination to eradicate *Helicobacter pylori* biofilm. They showed that the combinations CV-AMX, SHA-AMX, and CV-SHA had the highest antimicrobial and antibiofilm effects against *Helicobacter pylori* [66]. Mechmechan et al. revealed that the MIC of encapsulated CV (1.25 mg mL^−1^) was 4 times lower than that of free CV (5 mg mL^−1^) against *P*. *aeruginosa* [51]. Moreover, Ashrafudoulla et al. also revealed that CV could dramatically reduce the amount of *P. aeruginosa* biofilm [67].

The present study revealed that the combination of CV + Zn with CIP and TOB reduces their MIC by 5-fold and 4-fold compared to CIP and TOB alone, respectively. Furthermore, a consistent decreasing pattern in the bactericidal effect MBC of all three antibiotics was observed, particularly for AZM and TOB, with a significant 12- and 8-fold decrease, respectively. We showed that when CV + Zn was combined with CIP, the MBEC decreased to 0.156 and 0.007 mg/mL, resulting in a 64- and 4-fold reduction in concentration. Soumya et al. surveyed the antibiofilm properties of CV and thymol components against *P. aeruginosa* (ATCC 27853). Results obtained from their study revealed that doses of 2-MIC of CV produced a greater influence on biofilm formation (inhibition exceeded 90%) [68]. Ashrafudoulla et al. revealed that on the CBD biofilm device, 0.06% CV reduced the biofilm formation in *P. aeruginosa* by 5.04 log CFU/peg. They showed that using growth on a polypropylene surface, 0.06% CV reduced the biofilm formation by 4.79 log CFU/peg [67].

Elkhatib et al. investigated the antibiofilm effects of different antibiotic combinations with ZnSO_4_ against *P. aeruginosa.* They showed that combinations of antibiotics (fluoroquinolones and carbapenems) with ZnSO_4_ had the highest synergistic effects against *P. aeruginosa* biofilm and reduced the MIC value. However, their findings showed that a combination of aminoglycosides (gentamicin and TOB) and ceftriaxone increased the MIC value [36].

The treatment of infectious lung diseases can be greatly improved with the use of pulmonary drug delivery. Drug delivery through the pulmonary route has the unique advantage of no first-pass effect and high bioavailability, making it an effective way to deliver therapeutics directly to lung lesions [69]. Drugs can be delivered to the lungs directly through local inhalation to induce therapeutic effects. Local inhalation has a few advantages over other methods of administration, such as high concentrations, high permeability, and quick absorption of antibiotics at the site of infection. Additionally, it prevents the liver first-pass effect and decreases systemic exposure and toxicity of antibiotics [70]. Given these advantages, IT could be a better approach for treating both upper and lower respiratory tract infections like those caused by *Pseudomonas* spp., but further research is necessary to ensure its safety and efficacy [70]. Cresti et al. investigated the efficacy and toxicity of an antimicrobial peptide (SET-M33) in a model of endotoxin-induced pulmonary inflammation. They showed that IT administration of SET-M33 at 0.5, 2, and 5 mg/kg inhibited BAL neutrophil cell counts. Moreover, the results of their research revealed that IT administration of SET-M33 reduced pro-inflammatory cytokines, such as TNF-α, KC, IP-10, MIP-1α, and MCP-1 [71].

IT delivery in this study was used to establish a localized lung infection model and achieve high pulmonary drug concentrations for enhanced bacterial clearance. Drugs like CIP, AZM, and TOB were selected for IP, PO, and IT delivery based on their pharmacokinetics, pharmacodynamics, and clinical relevance to respiratory infections [11,72,73]. While IT delivery maximizes drug concentration at the infection site, it remains invasive, with potential risks such as respiratory distress and applicability of animal models [74,75]. Alternative routes, such as PO and IP administration, were explored. PO administration, though non-invasive and patient-friendly, may not achieve the required pulmonary concentrations for effective localized infection treatment [74]. IP injection, while useful for systemic distribution, showed lower efficacy compared to IT delivery, reinforcing the challenge of achieving sufficient pulmonary concentrations with systemic routes [76].

IT administration of CV + Zn and TOB in this study showed bacterial clearance, likely due to the higher local drug concentrations achieved compared to systemic routes. IT delivery minimizes systemic toxicity while prolonging drug retention in the lungs, leading to a more sustained therapeutic effect. In contrast, systemic administration often struggles to maintain pulmonary antibiotic levels above the MIC, limiting its effectiveness [47,77,78,79]. Clinical studies have confirmed that direct pulmonary delivery, such as inhaled TOB, achieves higher lung concentrations and better bacterial eradication than systemic administration [80,81,82,83]. However, it is important to note that the superior efficacy of IT administration in our study does not imply general superiority over other routes. The effectiveness of an administration method depends on the specific clinical context. Our results, reflected in Table 2, highlight the enhanced antibacterial activity of the combination treatment, demonstrating a synergistic effect in vitro as well. Although IT delivery was the most effective in this study, the PO route remains a viable option. Inhaled antibiotics may achieve high local lung concentrations with minimal systemic absorption, but challenges such as non-uniform drug distribution, airway irritation, and difficulties in reaching deeper lung regions may limit their effectiveness [77,80,84]. Furthermore, despite promising preclinical results, robust clinical trial data are still needed to confirm whether higher target-site concentrations through inhalation lead to improved clinical and bacteriological outcomes compared to PO or IP administration [77,81].

Our histopathological analysis revealed that treatments with TOB combined with CV + Zn and CV + Zn alone, administered via IT and PO routes, as well as AZM combined with CV + Zn administered orally, resulted in lower lung tissue involvement and fewer periluminal infiltrates compared to single treatments. Notably, the combination of TOB + CV + Zn administered intratracheally produced the most favorable outcome, with minimal lung tissue involvement and reduced inflammation, thus showing that such combinations can effectively manage *P. aeruginosa* lung infections in mice. While antibiotics alone led to moderate to high lung tissue involvement, combinations with CV + Zn reduced lung involvement and inflammation. The intratracheal route was the most effective in minimizing lung tissue damage, but the PO route may still be preferred for safety reasons. In animal models, CV has been found to relax tracheal smooth muscle, stimulate β2-adrenergic receptors, inhibit muscarinic and histamine receptors, and decrease tracheal responsiveness [85]. It has been revealed that CV reduces emphysema in chronic obstructive pulmonary disease (COPD) and increases the anti-inflammatory cytokines and expression of the genes forkhead box P3 (FOXP3) and interferon gamma (IFNγ) in a model of asthmatic mice [58,85]. Moreover, a previously published study on a model of asthmatic mice revealed that CV decreases the pro-inflammatory activity and modifies the expression of genes responsible for transforming growth factor beta (TGF-β) and interleukins 4 and 17 (IL-4 and IL-17) [58]. CV can be used as a therapeutic agent in acute respiratory distress syndrome (ARDS) due to its inhibition of the activation of nuclear factor kinase (NF-κB) and mitogen-activated protein kinase (MAPK) signaling pathways [58].

The combination of ZnSO_4_ + carvacrol (CV + Zn) with CIP and TOB reduced their MIC by 5-fold and 4-fold, respectively. Histopathological analysis showed that the TOB + CV + Zn combination administered intratracheally produced the best results, with minimal lung tissue damage and reduced inflammation. Both IT and PO routes, particularly with CV + Zn alone or combined with AZM or TOB, were most effective for managing lung infections. This study aimed not only to evaluate the synergistic efficacy of antibiotic combinations but also to assess how different administration methods influence therapeutic outcomes. The route of delivery plays a crucial role in optimizing treatment strategies, making it essential to understand its impact for better clinical decision-making. However, further studies are needed to optimize oral administration for effectiveness and maximize the safety of the IT route. Ideally, treatment should balance efficacy with patient tolerability, favoring fewer adverse effects when possible. Combinations of systemic and inhaled antibiotics have been recommended for optimal treatment. This does not suggest that systemic routes are ineffective but rather highlights the advantage of local delivery for lung infections.

## 4. Materials and Methods

### 4.1. Ethics Approval

This study was approved by the Ethics Committee of the School of Medicine, Ahvaz Jundishapur University of Medical Sciences, Ahvaz, Iran, with reference number IR.AJUMS.ABHC.REC.1402.010. This experiment was performed in accordance with the National Research Council (US) Committee for Guide for the Care and Use of Laboratory Animals, 8th edition [86], and the guidelines provided by the ethical committee of experimental animal care at Ahvaz Jundishapur University of Medical Sciences.

### 4.2. Bacterial Strain and Culture Media

The *P. aeruginosa* reference strain PAO1, obtained from the Department of Microbiology, School of Medicine, Ahvaz Jundishapur University of Medical Sciences, Ahvaz, Iran, and the Alberta Health Services Regions reference strain PAO1 were used in this study for work carried out in Canada. Both isolates had the same phenotypes in the assays. Bacterial strains were stored at −70 °C in Microbank vials as described by the manufacturer (proLab Diagnostics, Richmond Hill, ON, Canada). Luria–Bertani broth (LB, VWR Chemicals, Leicestershire, UK, Lot# 190756384) and Mueller–Hinton broth and agar (MHB, BD Bacto, Oxoid, Basingstoke, UK, Cat# X296B) were used as the growth medium and susceptibility testing media in this study [87,88].

### 4.3. Stock and Working Metal(loid)-Based Antibiotic (MBA) Solutions

Metal(loid)-based antimicrobial (MBA) zinc sulfate (ZnSO_4_·7H_2_O, Fisher Scientific Fair Lawn, NJ, USA, Lot# 723689) and plant-based compounds (PBCs), carvacrol (TCI, Portland, OR, USA, Lot# NDFYD-GT, >98%), along with three antibiotics (ciprofloxacin (CIP) (SIGMA, St. Louis, MO, USA, Lot# 146C0896), tobramycin (TOB) [89], and azithromycin (AZM)) were used [90]. Stock solutions for all antibiotics were prepared at a concentration of 10 mg/mL, while for all other agents, they were prepared at 200 mg/mL. Working solutions for antibiotics were made at 2 mg/mL, and for all other agents, they were made at a range from 2 to 40 mg/mL in distilled and deionized (dd) H_2_O. For PBCs and MBAs, they were used at a concentration of their MIC value for each agent alone under the same environmental conditions. All stock dilutions were stored in glass vials at room temperature in a dark place for no longer than 2 weeks. No more than 30 min before experimental use, working solutions were prepared from stock metal solutions by combining equal amounts of each media. Antimicrobial assays were performed in a 96-well plate (the challenge plate), and serial dilutions of each agent with a dilution factor of 2 were prepared; reservation of the first column served as a contamination control (media, 0 mg/mL antibacterial agents, and no bacteria), and the last column served as a growth control (media and bacteria, 0 mg/mL antibacterial agents, and with bacteria).

### 4.4. Planktonic Susceptibility

Minimum inhibitory concentration (MIC) and minimum bactericidal concentration (MBC) assessments were conducted on all bacterial strains using 96-well microtiter plates. To prepare the wells for bacteria, serial dilutions of the antibacterial agents were carried out along the rows of two microtiter plates. The antibacterials were 2-fold diluted with Mueller–Hinton broth (MHB) in each column, reaching a volume of 75 µL. The 1.0 McFarland standard was diluted 15-fold in MHB, and 75 µL of inoculum was added to the wells treated with antibacterials, resulting in a total volume of 150 µL. The microtiter plates were covered, then shake-incubated overnight at 150 rpm and 37 °C. MIC was determined based on visible bacterial growth. In cases of antibacterial opacity or ambiguous results, streaking of well cultures on Mueller–Hinton agar (MHA), overnight incubation, and subsequent comparison to controls were performed. MBC was determined by transferring 3 µL of culture from all MIC microtiter plate wells to 147 µL of MHB in fresh plates. These plates were shake-incubated overnight at 150 rpm and 37 °C, and bacterial growth was visually inspected [91].

### 4.5. In Vitro Assay for Cell Viability and Cytotoxicity

#### Cell Culture

Human lung fibroblast cells, MRC-5 (ATCC^®^ CCL-171™, Manassas, VA, USA), were purchased from the Pasteur Institute (Pasteur Institute of Iran (IPI), No. 69, Pasteur Ave, Tehran, Iran). MRC-5 cells, derived from normal human lung fibroblasts, are widely used in toxicology studies to evaluate the potential cytotoxic effects of antimicrobial agents on non-cancerous human cells. Their normal, non-transformed nature makes them a reliable model for assessing biocompatibility. IT administration is the route used to induce infection in mice, creating an animal model for respiratory infection. Therefore, we chose MRC-5 cells, which are human pulmonary fibroblasts, as the experimental cell line for this study. However, as normal epithelial cells are difficult to maintain and expand in culture, we selected this widely used cell line for our screening [92,93].

The cells were cultured in Dulbecco’s modified Eagle medium (DMEM) and Roswell Park Memorial Institute (RPMI) 1640 media containing 10% fetal bovine serum (Hyclone; Thermo Fisher Scientific, Waltham, MA, USA). Growth media contained 100 units/mL penicillin and 50 μg/mL streptomycin, respectively. The cells were maintained at 37 °C in a humidified atmosphere in the presence of 5% CO_2_. To determine and compare the cytotoxicity of the single compounds (PBC and MBA), 3-(4,5-dimethylthiazol-2-yl)-2,5-diphenyltetrazolium bromide (MTT) cytotoxicity assay was used according to a previously optimized method [94]. Briefly, human lung fibroblast MRC-5 cells were cultured in DMEM and RPMI 1640 medium containing 10% fetal bovine serum. Growth media contained 100 units/mL penicillin and 50 μg/mL streptomycin, respectively, and these cells were maintained at 37 °C in a humidified atmosphere of 5% CO_2_. The cells were seeded into 96-well culture plates at 1 × 10^4^ cells per well. The cells were incubated with the above cell culture medium (100 μL) containing each compound at a starting concentration of 5 mg/mL to 0.31 mg/mL for 24 h. Plates treated with the medium but without the agents were run in parallel and used as controls. Following treatment, the amount of formazan crystals formed was measured after 2 h of exposure to an MTT solution in phosphate-buffered saline, and absorbance values were measured at 570 nm by an enzyme-linked immunosorbent assay plate reader. Cytotoxicity experiments were performed in triplicate, and cytotoxicity results were calculated according to a previously described method [94]. All experiments were completed in triplicate (technical replicates) and repeated at least three different times.

### 4.6. Synergism Susceptibility Testing of Microbial Planktonic Growth

Similar to MIC and MBC for single antibacterial agents, serial dilutions of the antibacterial agents individually and in combination were carried out along the rows of three microtiter plates. Working solutions for antibiotics were prepared at concentrations ranging from 2 to 4 mg/mL (2–4 fold higher for combinations as these solutions were diluted by adding them to each other), and for all other agents, they were made at a range from 2 to 40 mg/mL in distilled and deionized (dd) H_2_O. The antibacterials were 2-fold diluted with MHB in each column, reaching a volume of 75 µL. The 1.0 McFarland standard was diluted 15-fold in MHB, and 75 µL of inoculum was added to the wells treated with antibacterials, resulting in a total volume of 150 µL. This protocol was based on the microdilution technique, where the drugs are diluted and combined together in several 96-well plates. In the first set of 96-well plates, MHB was added, followed by the first required drug (e.g., CV here) to serially dilute it. After the first step was completed, another set of 96-well plates was used to dilute the second drug (e.g., ZnSO_4_), which was transferred by removing a specific volume of drug 2 and added to the corresponding wells in the first set of 96-well plates that contained drug 1. The third step involved adding the required concentrations of the third drug (e.g., tobramycin) to the appropriate plates in the initial set containing combinations of drugs 1 and 2. Then, the bacterial inoculum was prepared and added to all wells in the plates. For each plate analysis, the same MIC and MBC steps indicated were conducted to survey the bacteriostatic, bactericidal, and synergistic effects of the combinations. The microtiter plates were covered, then shake-incubated overnight at 150 rpm and 37 °C. MIC was determined based on visible bacterial growth. In cases of antibacterial opacity or ambiguous results, streaking of well cultures on MHA, overnight incubation, and subsequent comparison to controls were performed. Synergistic MBC was determined by transferring 3 µL of culture from all MIC microtiter plate wells to 147 µL of MHB in fresh plates. These plates were shake-incubated overnight at 150 rpm and 37 °C, and bacterial growth was visually inspected [27,95].

#### Determination of FIC for the Detection of Synergism Effects

The synergistic interaction rules suggested by the American Society for Microbiology for the testing of planktonic cells are used for both MIC and MBC synergism data obtained here [96]. The fractional inhibitory concentration (FIC) and fractional bactericidal concentration (FBC) index for each combination of antimicrobial agents were calculated with the following formula:

FIC = MIC antibiotic A in combination/MIC antibiotic A alone + MIC antibiotic B in combination/MIC antibiotic B alone.

FBC = MBC antibiotic A in combination/MBC antibiotic A alone + MBC antibiotic B in combination/MBC antibiotic B alone.

To evaluate antimicrobial interactions, we used the lowest FIC/FBC index method as described by Bonapace et al. [97]. The lowest FIC/FBC obtained for all inhibitory or bactericidal combinations on the checkerboard was considered the FIC/FBC for the pair. Finally, FIC/FBC were interpreted as follows: FIC/FBC < 0.8 = synergy, FIC/FBC ≥ 0.8 and ≤ 1.2 = partial synergy, and FIC/FBC > 1.2 = antagonistic.

### 4.7. Biofilm Cultivation

Biofilms were grown in a Calgary biofilm device (CBD; commercially available as the MBEC physiology and genetics assay [Innovotech Inc., Edmonton, AB, Canada]), as originally described by Ceri et al. [98]. Starting from cryogenic stocks, *P. aeruginosa* PAO1 was streaked out twice on tryptic soy agar (TSA). Then, 150 µL of 1.0 × 10^7^ CFU/mL bacteria inoculum was transferred into each well of a 96-well microtiter plate, and the sterile peg lid of the CBD was inserted into the plate. The inoculated device was then placed on a microplate shaker at 150 rpm for 24 h of incubation at 37 °C and 95% relative humidity.

### 4.8. Determining the Minimal Biofilm Inhibition Concentration (MBIC)

All biofilm experiments were carried out using a Calgary biofilm device. Briefly, −70 °C stored bacteria were sub-cultured on MHA at 37 °C overnight (O/N) to obtain a pure single colony. 75 μL of the desired concentration of PBCs was added to 96 wells, 75 μL of bacteria (1.0 × 10^6^ CFU/mL) added in each well, and finally, the polystyrene CBD pegged lid was placed into the 96 wells and incubated for 48 h at 37 °C in a microplate shaker incubator at 150 rpm. The CBD lids were removed from the media, and the adhered biomass was rinsed two times with distilled water. The extent of the biofilm biomass was determined using a crystal violet assay, which allowed the minimum biofilm inhibitory concentration (MBIC) to be determined [99]. The wells with the highest dilution of agents but which had no bacterial biofilm and zero OD600 absorption were considered MBIC [18]. Results from at least three separate biological replicates are reported [100].

### 4.9. Minimum Biofilm Eradication Concentration (MBEC)

After developing a biofilm on CBD, the pegs were rinsed twice with 0.9% saline to wash away planktonic bacteria, then placed into a 96-well microtiter plate containing two-fold serial dilutions of the MBAs on the 150 μL of each media; a column was reserved for bacterial growth in the absence of the agents. The microtiter plate was then incubated for 24 h in a humidified incubator at 37 °C on a gyrorotary shaker at 150 rpm. This treatment was used to determine the minimum biofilm eradication concentration (MBEC) of each agent [101]. The last well, which had no bacterial biofilm and OD600 absorption, was considered MBEC.

### 4.10. Synergism High-Throughput Susceptibility Testing of Microbial Biofilm Growth

“Checkerboard” arrangements of combinations were made in 96-well microtiter plates as previously described [102]. When prepared, each checkerboard microtiter plate had one column as a negative control (just media without bacteria and agents) and one column growth control as a positive control (without agents, with media, and bacteria). It would also contain 10 different concentrations of each agent alone and each compound and antibiotic at different combinations of concentrations, leading to the binary concentration array in the checkerboard. For each checkerboard analysis, the same MBIC and MBEC steps as described before [103] were conducted to survey biofilm eradication, prevention, and synergism potency of combinations.

#### Fractional Biofilm Inhibition and Eradication Concentration (FBIC/FBEC)

The synergistic interaction rules suggested by the American Society for Microbiology for the testing of planktonic cells were used here for MBEC synergism data obtained [104]. The fractional biofilm eradication concentration (FBIC/FBEC) index for each combination of antimicrobial agents was calculated with the following formula:

FBIC/FBEC = MBIC, MBEC antibiotic A in combination/MBIC, MBEC antibiotic A alone + MBIC, MBEC antibiotic B in combination/MBIC, MBEC antibiotic B alone.

To evaluate antimicrobial interactions, we used the lowest FBEC index method as described by Bonapace et al. [97]. The lowest FBEC obtained for all eradication combinations on the checkerboard was considered the FBEC for the pair. Finally, FBEC was interpreted as follows: FBIC/FBEC <0.8 = synergy, FBIC/FBEC ≥0.8 and ≤1.2 = partial synergy, and FBIC/FBEC >1.2 = antagonistic.

### 4.11. In Vivo Experiments (PAO1 Respiratory Infection in a Murine Model)

Thirty-three pathogen-free BALB/c mice (6–8 weeks and 18–25 g) were purchased from the Laboratory Animal Unit of Ahvaz Jundishapur University of Medical Sciences (Ahvaz, Iran). These mice were divided into 11 groups: non-infected control, infected control, AZM-treated (PO), AZM + CV + Zn-treated (PO), CIP-treated (IP), CIP + CV + Zn-treated (IP), TOB-treated (IT), TOB + CV + Zn-treated, and CV + Zn-treated groups (IT, IP, and PO). All mice were treated with MIC and sub-MIC concentrations of antibacterials. The mice were maintained in micro-isolator cages in a pathogen-free barrier facility throughout the experiment, being provided with food and water ad libitum for 10 days to acclimatize to the environment. An experimental model of chronic lung infection was established as described previously with minor modifications [105,106,107]. Briefly, mice were anesthetized by intraperitoneal injection of 60 mg/kg of body weight ketamine (Pfizer) and 5 mg/kg xylazine (Sigma-Aldrich, Madrid, Spain) and instilled intratracheally [108] with ~ 5 × 10^5^ CFU PAO1 [109] in 50 mL PBS. Antibacterial treatments were started at 24 h post-infection (0.05 mg/kg in 0.5 mL of PBS) and administered once a day for three consecutive days [89]. Control mice were instilled with 100 µL of saline or PBS without antibacterial agents.

Antibacterial treatments were initiated 24 h post-infection and administered daily for three consecutive days. The daily administration of antibacterial treatments was as follows: 200 µL for intraperitoneal (IP), 30 µL for IT (intratracheal inhalation), and 250 µL for PO (oral) routes. Treatments included TOB (IT), CIP (IP), AZM (PO), CV + Zn (PO, IT, and IP), and combinations thereof. Control groups received sterile saline USP (0.9% sodium chloride) via IP administration. The specified volumes were administered via the mentioned routes for each mouse.

#### 4.11.1. Blood and Lung Collection

At the end of the experimental period, 24 h after the last dose, all rats were anesthetized with chloroform (Merck, Darmstadt, Germany). Blood sampling was performed via the cardiac puncture through the diaphragm to prevent any damage to the lungs. Blood samples were collected into sterile tubes with and without anticoagulant (EDTA). Whole blood samples (with EDTA) were subjected to hematologic assessment. In some cases, when the amount of blood was insufficient for those assays, blood from two or more mice from the same group was pooled.

For assessing bacteria by colony-forming unit (CFU) enumeration, lung samples were weighed and homogenized in 1 mL PBS and serially diluted (lung samples 1:10, 1:100, 1:1000, and 1:10,000). Then, 30 mL of each dilution was plated on a 30° angle to form stripes. All plates were incubated at 37 °C. Cultures were counted, and CFUs were calculated taking the weight of the organ into account. Results were counted logarithmically for each group and were compared with the control group.

#### 4.11.2. Hematologic Assessment

The total erythrocyte count (RBC) and total white blood cell count (WBC), including subcategories, were measured using the BC-2800Vet hematology analyzer (Mindray, Shenzhen, China) at the Veterinary Faculty of Shahid Chamran, University of Ahwas.

#### 4.11.3. Histopathological Studies

One lung was removed under aseptic conditions and kept in 10% buffered formalin for 24 h. Then, the lung tissue was dehydrated, kept in xylene, and processed in paraffin. Sections were cut at 5–10 μm thickness and stained with hematoxylin and eosin. Histological evaluation was done by a pathologist in a blinded fashion.

### 4.12. Statistical Analysis

GraphPad Prism 8 (GraphPad Software, Inc., La Jolla, CA, USA) was used for the statistical analyses. Statistical analyses of the data for in vivo tests were conducted using one-way ANOVA with ordinary and Tukey’s multiple comparison tests. *p*-values <  0.05 were considered statistically significant.

## Figures and Tables

**Table 1:** Antimicrobial levels of the compounds investigated here against the *P. aeruginosa* reference strain PAO1.

Agents	MIC (mg/mL)	MBC (mg/mL)
Carvacrol (CV)	10	20
Zinc sulfate (ZnSO_4_)	2.5	5
Ciprofloxacin (CIP)	0.001	0.007
Azithromycin (AZM)	0.007	0.5
Tobramycin (TOB)	0.00025	0.003

**Table 2:** The synergistic planktonic antibacterial properties of carvacrol (CV) and zinc sulfate (ZnSO_4)_ in combination with the three antibiotics.

Agent A	Agent B	MIC/MBC (mg/mL) *				FIC/FBC Index **	Outcome
		Alone A	Alone B	Combination A	Combination B		
Carvacrol (CV)	Ciprofloxacin (CIP)	10/20	0.001/0.007	0.009/0.078	0.0002/0.003	0.2/0.4	Synergistic/synergistic
Zinc sulfate (ZnSO_4_)	Ciprofloxacin (CIP)	2.5/5	0.001/0.007	0.01/0.156	0.0005/0.007	0.5/1	Synergistic/partially synergistic
**Ciprofloxacin (CIP)**	**CV + Zn**	**0.001/0.007**	**1.25/2.5**	**0.0002/0.003**	**0.009/0.078**	**0.2/0.4**	**Synergistic/synergistic**
Carvacrol (CV)	Azithromycin (AZM)	10/20	0.007/0.5	0.078/5	0.003/0.25	0.4/0.7	Synergistic/synergistic
Zinc sulfate (ZnSO_4_)	Azithromycin (AZM)	2.5/5	0.007/0.5	0.78/2.5	0.003/0.125	0.4/2	Synergistic/antagonistic
Azithromycin (AZM)	CV + Zn	0.007/0.5	1.25/5	0.015/0.0625	0.313/1.25	2.2/1.8	Antagonistic
Carvacrol (CV)	Tobramycin (TOB)	10/20	0.00025/0.003	0.009/0.009	0.00025/0.00025	1/0.08	Partially synergistic/synergistic
Zinc sulfate (ZnSO_4_)	Tobramycin (TOB)	1.25/5	0.00025/0.003	0.009/0.01	0.00025/0.0005	1/0.1	Partially synergistic/synergistic
**Tobramycin (TOB)**	**CV + Zn**	**0.00025/0.003**	**1.25/2.5**	**0.0006/0.00025**	**0.004/0.009**	**0.2/0.08**	**Synergistic/synergistic**

* Lower MIC/MBC shows better antibacterial effects. ** Lower FIC/FBC index shows better Synergism effects. (<0.8 = synergy, ≥0.8 and ≤1.2 = partially synergy, and >1.2 = antagonistic).

**Table 3:** Synergistic biofilm eradication efficacy of CV and ZnS in combination with three antibiotics (CIP, AZM, and TOB).

Agent A	Agent B	Biofilm Eradication (mg/mL) *				FBEC Index **	Outcome
		Alone A	Alone B	Combination A	Combination B		
Carvacrol (CV)	Ciprofloxacin (CIP)	20	0.0312	0.625	0.0312	1.03	Partially synergistic
Zinc sulfate (ZnSO_4_)	Ciprofloxacin (CIP)	5	0.0312	0.625	0.0312	1.1	Partially synergistic
**Ciprofloxacin (CIP)**	**CV + Zn**	**0.0312**	**10**	**0.007**	**0.156**	**0.2**	**Synergistic**
Carvacrol (CV)	Azithromycin (AZM)	20	2	5	0.25	0.3	Synergistic
Zinc sulfate (ZnSO_4_)	Azithromycin (AZM)	10	2	5	0.25	0.6	Synergistic
Azithromycin (AZM)	CV + Zn	2	10	0.125	2.5	0.4	Synergistic
Carvacrol (CV)	Tobramycin (TOB)	20	0.125	1.25	0.625	0.5	Synergistic
Zinc sulfate (ZnSO_4_)	Tobramycin (TOB)	2.5	0.125	0.625	0.0312	0.4	Synergistic
Tobramycin (TOB)	CV + Zn	0.125	2.5	0.0312	0.625	0.5	Synergistic

* A lower MBEC indicates greater biofilm eradication efficacy. ** Lower FBEC index shows better synergism effects. (<0.8 = synergy, FBEC ≥0.8 and ≤1.2 = partial synergy, and FBEC >1.2 = antagonistic).

**Table 4:** Assessment of lung tissue involvement, alveolar air space, pneumonitis score, and inflammatory scores across treatment groups.

Groups	Percentage of Alveolar Air Area (%)	Pneumonitis Score (Alveolar/Interstitial)	Periluminal Infiltrates Score (Around Airways/Vessels)	Percentage of Lung Tissue Involved (%)
Control	80	0	0	0
Bacteria	10	3	3	80
CV + Zn (IP *)	55	2	2	45
Ciprofloxacin + CV + Zn (IP)	65	1	0.8	35
Ciprofloxacin (IP)	55	2	0.9	45
Tobramycin (IT)	50	2	2	60
CV + Zn (IT *)	65	1	0.5	8
Tobramycin + CV + Zn (IT)	70	1	0.2	5
Azithromycin (PO *)	60	2	1	55
Azithromycin + CV + Zn (PO)	65	1	0.7	30
CV + Zn (PO)	65	1	0.7	10

* IT: intratracheal inhalation; IP: intraperitoneal; PO: oral.

## Data Availability

All data supporting the findings of this study are provided in the manuscript, including tables and figures. No additional datasets were generated or analyzed during this study.

## Abbreviations

The following abbreviations are used in this manuscript:

ZnSO_4_	zinc sulfate
CV	carvacrol
CIP	ciprofloxacin
TOB	tobramycin
AZM	azithromycin
MIC	minimum inhibitory concentration
MBC	minimum bactericidal concentration
FIC	fractional inhibitory concentration
FBC	fractional bactericidal concentration
MBIC	minimum biofilm inhibition concentration
MBEC	minimum biofilm eradication concentration
PBC	plant-based natural compounds
MBA(s)	metal(loid)-based antimicrobials
IT	intratracheal
IP	intraperitoneal
PO	oral
MRC-5	human lung fibroblast cells
MTT	3-(4,5-dimethylthiazol-2-yl)-2,5-diphenyltetrazolium bromide
CFU	colony-forming unit
WBC	white blood cell
RBC	red blood cell
WHO	World Health Organization
COPD	chronic obstructive pulmonary disease
PBS	phosphate-buffered saline
FOXP3	forkhead box P3
IFNγ	interferon gamma
TGF-β	transforming growth factor beta
NF-κB	nuclear factor kappa B
IL	interleukin
MAPK	mitogen-activated protein kinase
ARDS	acute respiratory distress syndrome
CBD	Calgary biofilm device
